# What Is Behind the Buzzword for Experts and Laymen: Representation of “Artificial Intelligence” in the IT-Professionals’ and Non-Professionals’ Minds

**DOI:** 10.5964/ejop.5473

**Published:** 2022-05-31

**Authors:** Paweł Fortuna, Oleg Gorbaniuk

**Affiliations:** 1 Institute of Psychology, John Paul II Catholic University of Lublin, Lublin, Poland; Università Cattolica del Sacro Cuore - Milan, Italy

**Keywords:** mental representation, expertness, IT-professionals, artificial intelligence

## Abstract

The aim of the presented research was to define the differences between information technology (IT) professionals (ITP) and non-professionals (NP) in the way of understanding artificial intelligence (AI). The research was designed in the tradition of categorization research. In an online study participants were asked to make typicality and familiarity judgments for 50 AI exemplars. Two types of analyses were carried out, which made it possible to identify and compare the hierarchy of AI designates (graded structure) and the dimensions of their groupings. We have found that “invisible AI” exemplars were highly rated by ITP, but “visible AI” by NP. Expert knowledge allows ITP to systematize AI exemplars based on both structural and functional elements. On the other hand, laymen indicate the functions that AI-driven products perform, rather than their structures. For ITP, they are primarily algorithmic systems, while for NP they are systems that emulate the functions of living organisms.

AI is currently one of the most fashionable and ambiguous terms. It is said that “AI applies to…” “AI can…,” “AI threatens…,” but what does it actually mean? At the individual level, the concept of AI is a mental representation that refers to a set of designates (exemplars of a category) and includes their essential properties (Smith & Medin, 1981). The content of the representation depends on the experience of the individual and the context in which it is acquired and applied, which makes it different for experts and novices (Murphy & Medin, 1985). Until now, no systematic research has been conducted into representations of AI but the problem is not being ignored. The source of data on this subject are mainly the surveys, which indicate that for the respondents AI is mainly perceptually identified artifacts, such as humanoid robots (Davies, 2020). These studies allow to capture the most mentally accessible exemplars of categories, but they do not provide insight into how people arrange designations within the category and ignore individual differences in understanding AI, treating the respondents as a homogeneous group.

The research presented in this article intends to fill this gap by defining how information technology professionals (ITP) and NP deal with the transformation of informational “chaos” about AI into a subjective “cosmos.” Both cognitive and practical reasons decided to differentiate between these groups. First, due to the qualifications of ITPs, they meet the criterion of expertise, with its indicator in the form of structured and usable specialist knowledge (Farrington-Darby & Wilson, 2006). Second, the importance of ITPs in the modern world grows as they are the ones who give direction and impetus to digital transformation. Third, the social role of ITP as people building a bridge between developers and users of AI-based solutions is changing. Due to the need to work closely with people who do not have an IT education, the expectations of employers regarding the ITP competence profile are constantly changing (Patacsil & Tablatin, 2017). An IT-job today has the “hybrid” form because it requires a multidimensional skill set combining technology skills and human focus (Bullen, Abraham, Gallagher, Kaiser, & Simon, 2009). Programmers and administrators less and less often resemble stereotypical nerds who have been described as “intelligent but anti-social people” (Ward, 2014). Such an unfavorable image has been perpetuated for years by media messages, in which a computer expert is most often a man with admirable cognitive attributes, but still socially awkward or even avoiding people altogether (Wong, 2019). Although this stereotype is reproduced in popular culture (Mendick & Francis, 2012), it is no longer widely accepted among young people who understand that IT experts must develop both “hard” (technical) and “soft” (non-technical) skills (Wong, 2019).

The theoretical basis of the presented research is the theory of prototypes (Rosch, 1973) and the theory of concepts as naive theories (Medin & Wattenmaker, 1987). The theory of prototypes is useful in relation to areas that are difficult to define, because it is based on the observation that the concepts, that people use in everyday life, do not have sharp boundaries (Dean, 2003). According to it, man cognitively orders the world by prioritizing the items in terms of their typicality as examples. On the other hand, the idea of concepts as naive theories indicates that people flexibly select the dimensions on which they arrange objects in the psychological space. Identifying the hierarchy of AI designates and the dimensions of their grouping brings us closer to grasping how people understand this part of their world. In turn, capturing the differences in this area between ITP and NP facilitate the design of activities, supporting better cooperation between the two groups.

## Difficulty with Defining AI

The introduction of the name AI (McCarthy, Minsky, Rochester, & Shannon, 1955) was also the beginning of the discussion on the definition of this concept (Nilsson, 2009). Although there is a general consensus that AI is the attempt “to make a computer work like a human mind” (Wang, Liu, & Dougherty, 2018, p. 2), at the more detailed levels of analysis we deal with terminological “tower of Babel.” The very concept of "intelligence" is problematic. In the area of AI research, over 28 definitions have been developed in the last decade and subsequent attempts to systematize the approaches are still lively discussed (Monett, Lewis, & Thórisson, 2020). Regardless of the debate over the concept of intelligence, AI develops as “…a scientific study of what problems can be solved, what tasks can be accomplished, and what features of the world can be understood computationally (i.e., using the language of Turing Machines), and then to provide algorithms to show how this can be done efficiently, practically, physically, and ethically” (Rapaport, 2020, p. 54). Many subdomains are developing within it, and exemplars of the concept of AI can be classified at various levels of generality. At the general level, Russell and Norvig (2016), classify intelligent agents on the basis of program design, and at a more detailed level, according to their functions, distinguishing systems specialized in solving various types of problems. Some of them are softbots, while others are robots endowed with effectors to perform tasks by manipulating objects in physical world. The number of areas in which AI is used is growing, which only widens the range of possible meanings of this concept. On a detailed level, we can indicate applications used by the governments and military, transportation, information searching, art, communication, education, entertainment, finance, industry, medicine, science, security, services, etc. In addition, many of the new “intelligent tools” operate under marketing names, for example, Alexa or Siri.

## Acquisition of AI Representation

Most people encounter the notion of AI and portraits of AI on a daily basis in popular culture. The frequency and scope of direct contacts with intelligent agents depend on the habits of using technology and the professional specialization. Surveys show that people who contact AI-based products are not always aware of it (Brown, 2017). It is considered, that social awareness of AI seems to depend on the visibility of its application (Davies, 2020). It found that 90% of respondents are aware that the voice assistant (tangible device qualified as “visible AI”) is AI-based, while only one in three has associated AI with online shopping sites, video streaming services and social media (algorithms qualified as “invisible AI”). The obtained results are consistent with the observations collected in the British research (The Royal Society, 2018), which revealed the limited knowledge of respondents about AI: only 9% know the term “machine learning.” Polish respondents, on the other hand, asked to spontaneously provide examples of AI most often mentioned a humanoid robot, a technologically advanced computer, and self-driving car (AV) (Maison, 2019). It has been found that for the average Pole, AI is a phenomenon remote from everyday life, although as many as 81% believe that they know what it is, but only 17% of them believe that AI is committed to innovative smartphones.

The fact that the respondents indicated “visible AI” exemplars as typical examples of AI is probably the result of the influence of media messages. AI-narratives present in pop culture include “…portrayals of any machines (or hybrids, such as cyborgs) to which intelligence has been ascribed, which can include representations under terms such as robots, androids or automata” (Cave, Dihal, & Dillon, 2020, p. 5). Some of the narratives about AI are non-fictional (e.g., TV news), and some are fictional messages (e.g., sci-fi movies). An analysis of the content of articles on AI, published in the New York Times in 1985–2016, shows that their subject matter has changed: from space weapons, through chess, search engines, to AV (Fast & Horvitz, 2017).

## IT-Professionals as Experts

Participants are classified as experts in expertise research most often on the basis of their qualification (Farrington-Darby & Wilson, 2006). The ITP meet this criterion, although they differ in terms of specialization, experience and position (e.g., junior, mid-level and senior developer). The report of the *IT Community Survey 2020* conducted in Poland shows that the ITPs are educated and development-oriented people. They obtain additional specialist information mainly from blogs and articles, online courses and books, and the greatest source of motivation for them is the desire to self-improve (70%). Knowledge of new technologies and experience resulting from contact with innovations distinguish ITP from NPs. The most important skills that characterize experts include perceiving meaningful patterns in their domain and representing a problem at a deeper level than novices Chi, Glaser and Farr (1988). Shanteau (1992) highlights extensive and up to date content knowledge and sense of what is relevant when making decisions. In turn, according to Cellier et al. (1997) experts have more complete representations of the task domains, a more global and functional view of a situation and take a wider range of data into account in diagnosis. Research shows that experts differ from laymen in both the amount and type of information they have, and different types of experiences with a taxonomic category influence the internal structure of the category (Bailenson, Shum, Atran, Medin, & Coley, 2002).

## Current Study

The aim of our research was to determine the differences between ITP and NP in the way of understanding AI by comparing the hierarchy of AI designates (graded structure) and the dimensions of their grouping. The research is exploratory in nature, so no hypotheses have been formulated. The justification for the adopted research concept is presented below.

### Typicality and Graded Structure of Category

As previously shown, currently there is no unambiguous definition of AI, because it is not possible to indicate one ontological basis of AI. However, such concepts are rare in human cognition, and people use almost exclusively concepts with blurred boundaries, when creating a colloquial representation of the world (Gluck & Bower, 1988). In line with the prototype concept and other similarity-based models (Smith & Medin, 1981), categories exhibit graded structure, in which some exemplars represent the category better than others, and there is a stable within-category structure, usually described as the typicality gradient. Typicality reflects how representative an exemplar is for a category (Hampton, 2007). The attribution of typical features to a given specimen depends on the experience of the individual (Murphy & Medin, 1985) and the level of expertise (Bailenson et al., 2002). In this study we used the method of determining typicality, which consists in presenting the participants with the referent of the category and instructing them to evaluate *goodness-of-example* (GOE), delineated in Rosch and Mervis, 1975. As people differ in the level of knowledge of the designations of the concept of AI, apart from the GOE assessment, they also estimated the degree of familiarity. This term was also used to confirm the differences between ITP and NP in terms of expertise.

### Dimensions of AI Exemplars Grouping

The category of AI is diverse and every day brings information about new innovations. A person who wants to reduce such a variety of stimuli cannot refer to a normative systematization as for example when he group elements in the Mendeleev table. The mental “taxonomic sort of exemplars” must therefore be based on subjectively defined dimensions that explain the similarities and differences between the categorized objects. Rosch (1978) distinguished two dimensions of conceptual representations (vertical and horizontal), however the studies comparing experts and laymen show that these dimensions are not universal. Johnson and Mervis (1997) found that experts used information from different levels, while novices only duplicated information from the basic level. This is in line with the notion of concepts as naive theories, according to which concepts should be treated not as isolated mental representations, but as part of the individual’s knowledge that is influenced by them (Medin & Wattenmaker, 1987). The idea of concepts as naive theories makes it possible to predict differences in the dimensions used by ITP and NPs, due to different knowledge about AI and experience in contact with exemplars of this category.

## Method

### Subjects

Due to the fact that the analyses were to be carried out separately for the ITP and NP groups, the same size of both samples was not forced. At the same time, due to the fact that the stability of the factor structure depends on the size of the sample, the largest possible samples were recruited for the study. Accessibility to ITP was weaker, therefore the number of participants in this group is lower than in NP.

#### IT-Professionals

One hundred seventy-three ITP (15.6% female, *M*_age_ = 34.7, range: 19–65 years) participants took part in the study; all participated voluntarily. The gender distribution, age and education were controlled according to survey data obtained in surveys on the Polish IT community (Ministerstwo Rozwoju Polski & Polski Towarzystwo Informatyzne, 2020). Participants were recruited via advertisements placed in internet IT groups in social networks and discussion boards and at intranet of various IT companies.

#### Non-Professionals

Three hundred thirty-eight NP (42.0% male, *M*_age_ = 30.7, range: 16–85 years) voluntaries participated in the study. They were recruited via advertisements placed in various internet groups in social networks.

### Materials

The research used a set of 50 AI exemplars (e.g., humanoid robot, robotic animal, book writing program). Their selection was based on: studies providing comprehensive AI kits; results obtained after entering the terms “AI” and “AI-driven products” into search engines; results of surveys; information contained in websites devoted to AI-related issues; YouTube videos; and AI-popularizing books. Each exemplar was presented to the participants in the form of a verbal label and a photo. The names of the examples were given the way they appear in popular messages. In the preliminary tests, three illustrative photos were selected for each exemplar, which appeared in search engines after entering its name. These photos were assessed by a group of students (*N* = 35), whose task was to indicate the one that best described the exemplar. Illustrations selected by the majority of respondents were selected for the study. The table with the names of the exemplars being assessed, and photos given to the subjects are listed in Appendix A.

### Procedure

An online study was conducted in May and June 2020 with the use of the Google Forms platform. Participants made typicality judgments for each of the 50 AI exemplars by GOE ratings (Rosch & Mervis, 1975). This instruction told participants to assess how good the example of the AI category was on a 7-point scale, ranging from 1 for *very poor example* to 7 for *very good example*. In addition, familiarity ratings of each AI example were collected. Participants rated their knowledge on a seven-point scale, with 1 meaning *unfamiliar* and 7, *familiar*. After typicality rating task and judgment of familiarity of each AI example respondents were asked to assess their general self-knowledge about AI on 5-point Likert scale, with 1 *I am a layman* and 5 *I am an expert*. Finally, they provided their gender, age, education, and professional background and were thanked for their contribution.

### Statistical Analysis

Two separate types of analyzes were performed in ITP and NP sample. In order to identify and compare the hierarchy of AI designates (graded structure) we applied t-test and Mann-Whitney’s test. Principal component analysis (PCA) with Varimax rotation was performed to determine the dimensions of AI exemplars grouping in each sample.

## Results

### Perceived Expertise of Participants

Participants were assigned to the ITP and NP groups on the basis of the information they provided about their occupation, but it was also checked whether there were differences in subjectively perceived specialist knowledge between these groups. The assessments of the knowledge of AI referents for all respondents were averaged (Cronbach’s α_ITP_ = 0.97 and α_NP_ = 0.97), and then they were compared between the groups. Statistically significant differences were found between the groups, *t*(509) = 2.91, *p* < .01, effect size Cohen’s *d* = 0.26. The ITP’s scores for the knowledge of the exemplars (*M* = 4.43, *SD* = 1.21) were higher than those of the NP (*M* = 4.11, *SD* = 1.17). The self-assessment of knowledge about AI of the two groups was compared and statistically significant differences were also found (Mann-Whitney’s test *z* = 6.09, *p* < .001, effect size *r* = .27). ITP assessed their knowledge of AI significantly higher (*M* = 3.17, *SD* = 0.82) than NP (*M* = 2.64, *SD* = 0.91). Additionally, a positive correlation was found between the ratings of familiarity and self-knowledge in both groups (τ-c_ITP_ = 0.30, *p* < .001 and τ-c_NP_ = 0.29, *p* < .001).

### Typicality and Graded AI Category Structure

The GOE scores of all subjects were averaged to obtain an overall typical index (Cronbach’s α_ITP_ = 0.96 and α_NP_ = 0.95). The comparison of these indicators showed significant differences—there was a lower overall assessment of the typicality of AI designates in the ITP group than in the NP group, *M*_ITP_ = 4.58 (*SD* = 1.09); *M*_TP_ = 4.95 (*SD* = 0.93); *t*(509) = 3.98, *p* < .001; effect size Cohen’s *d* = 0.36. Moreover, in the NP group, a significant positive correlation was found between the overall rating of typicality and the rating of familiarity with the items, *r*(338) = .35, *p* < .001. A similar relationship was not observed for the ITP group, *r*(173) = .05, *p* = .494, because the difference between correlation coefficients was statistically significant: *z* = 3.36, *p* < .001.

The graded structure of AI category has been established based on GOE ratings. The item with the highest average GOE grade received rank 1. Table 1 lists the ranks for all assessed AI representatives in the compared groups along with the average GOE and familiarity scores and the difference in ranks for each object (a positive value means that the item has a higher rank in the ITP category than NP). It was found that the most typical exemplar of the category in both groups was AV, although its GOE score in the ITP group was significantly higher than in the NP group (*M*_ITP_ = 6.12, *M*_NP_ = 5.88, *U* = 25,640, *p* < .05). Similarly, the ITP group had a significantly higher AV familiarity rate than the NP group (*M*_ITP_ = 5.42, *M*_NP_ = 4.72, *U* = 22,968, *p* < .001). There were no significant differences in both groups between the GOE rating of this object and the next AI example in the ranking. However, differences were noticed with regard to the type of successive AI objects with a lower rank than AV. In the ITP group they were (in descending order): facial recognition system, neural network and system for debating, and in the NP group: police robot, humanoid robot that creates images and social robot. Similarly, differences between groups were noted for objects with the lowest GOE scores. In the case of ITP, VR was the least typical example of the category, and in the case of NP, the product recommendation software (*M* = 3.79). The GOE VR score was significantly lower for ITP than for NP (*M*_ITP_ = 2.50, *M*_NP_ = 4.07, *U* = 16,890, *p* < .001). ITP rated GOE product recommendation software higher than NP (*M*_ITP_ = 2.50, *M*_NP_ = 4.07, *U* = 16,890, *p* < .001).

**Table 1:** Comparison of Typicality and Familiarity Assessments of the AI Exemplars for IT-Professionals (ITP) and Non-Professionals (NP)

AI Category Exemplars^a^	ITP			NP			Rank Differences
	GOE Ranks^b^	GOE Ratings^c^	Familiarity Ratings	GOE Ranks	GOE Ratings	Familiarity Ratings	
AV	1	6.11	5.42	1	5.88	4.72	0
Facial recognition system	2	5.94	5.43	5	5.71	4.90	−3
Neural network	3	5.93	5.11	43	4.44	3.45	−40
System for debating	4	5.73	3.31	9	5.46	2.86	−5
Strategy game program	5	5.55	4.30	18	5.12	3.87	−13
Risk determining program	6	5.50	4.08	14	5.28	3.63	−8
Skynet/Terminator	7	5.46	5.28	30	4.88	4.18	−23
Humanoid robot that creates images	8	5.44	3.90	3	5.83	3.49	5
Chess program	9	5.39	5.40	6	5.57	5.14	3
Chatbot	10	5.39	5.46	7	5.53	5.19	3
Police robot	11	5.32	3.86	2	5.84	3.69	9
Behavioral biometrics	12	5.28	4.73	8	5.52	3.75	4
Go playing program	13	5.28	4.37	21	5.05	3.66	−8
Press articles writing program	14	5.20	3.92	17	5.14	3.51	−3
Social robot	15	5.19	4.30	4	5.72	4.28	11
Poem-writing software	16	5.11	3.69	26	4.96	2.93	−10
Social bot	17	5.10	4.26	22	4.98	3.97	−5
Book writing program	18	5.03	2.86	16	5.19	2.57	2
Face generating program	19	4.97	4.09	32	4.78	3.37	−13
Virtual therapist	20	4.97	3.66	25	4.96	3.08	−5
Music generation program	21	4.94	4.32	29	4.89	4.30	−8
Image generation algorithm	22	4.92	4.06	39	4.63	3.50	−17
Financial service software	23	4.91	3.54	24	4.97	3.21	−1
Portrait-drawing robot	24	4.90	4.06	12	5.34	3.89	12
Humanoid robot	25	4.90	4.23	10	5.42	4.06	15
Program reconstructing images	26	4.88	3.50	11	5.34	3.18	15
Face replacing software	27	4.86	4.85	45	4.23	4.13	−18
Medical diagnostic software	28	4.62	3.83	17	5.12	3.81	11
Text translation program	29	4.57	5.80	34	4.75	6.11	−5
Video recommendation algorithm	30	4.38	5.69	42	4.46	5.87	−12
License plate recognition software	31	4.23	5.47	37	4.69	5.15	−6
Space robot	32	4.17	4.75	13	5.30	4.91	19
Intelligent teaching/learning support system	33	4.16	3.00	33	4.78	3.61	0
UAV	34	4.13	4.80	31	4.81	4.57	3
Product recommendation software	35	4.08	5.59	50	3.79	5.63	−15
Robotic animal	36	4.02	4.40	35	4.74	4.20	1
AR	37	3.98	4.89	38	4.68	4.09	−1
Nanobot	38	3.97	4.24	20	5.10	3.84	18
Robotic child	39	3.94	2.89	40	4.54	2.82	−1
Cyborg	40	3.88	4.54	15	5.22	4.65	25
Car navigation	41	3.75	6.16	27	4.93	6.29	14
Robot/android priest	42	3.65	2.13	46	4.17	1.99	−4
Sexbot	43	3.61	3.73	48	4.13	3.49	−5
Military robot	44	3.58	4.46	41	4.49	4.31	3
Vacuum cleaning robot	45	3.52	5.36	44	4.33	5.67	1
IoT	46	3.40	5.20	23	4.97	4.45	23
Surgical robot	47	3.23	4.05	28	4.89	4.46	19
COVID-19 contact tracing apps	48	2.88	3.82	47	4.15	3.11	1
Cobot	49	2.70	5.23	36	4.71	4.74	13
VR	50	2.50	5.34	49	4.07	5.00	1

*Note*. AI = artificial intelligence; AV = autonomous vehicle; UAV = unmanned aerial vehicle; AR = augmented reality; IoT = internet of things; VR = virtual reality; GOE = goodness-of-example.

^a^AI exemplars are listed in GOE ranks for ITP order. The table presents the short names of the items being assessed. ^b^GOE ratings were averaged across all participants for each exemplar of AI, and the GOE ranks and mean GOE ratings are presented in the table. ^c^Averaged familiarity ratings for each object are presented here.

### Dimensions of AI Exemplars Grouping

In order to determine the dimensions of the perception of psychological space, items of the AI category PCA with Varimax rotation were employed, counted separately in the ITP and NP group. A determinant of the correlation matrix for ITP and NP groups were respectively 4.71 × 10^-17^ and 2.20 × 10^-13^. KMO measure of sampling adequacy was .916 and .926, whereas Bartlett’s sphere test also proved to be considerably significant for ITP, χ^2^(1,225) = 5808.25, *p* < .001, and NP, χ^2^(1,225) = 5808.25, *p* < .001, groups. The eigenvalues of the first 10 non-rotated components in the ITP and NP group indicate that, according to the test scree, the optimal solution in both groups will be a 3-factor solution. It explains 50.7% (ITP) and 46.1% (NP) of the input variance, respectively.

In order to assess the degree of similarity between the factor structures (see Table 2) obtained in both groups, Tucker’s coefficients of congruence were computed, which is a measure of similarity between factor loadings. The highest congruence coefficients for the pairs of factors of both structures were as follows: .74 (between I ITP and III NP components), .91 (between II ITP and II NP components), .83 (between III ITP and I NP components). In the light of the criteria proposed by Lorenzo-Seva and ten Berge (2006), the obtained Tucker’s coefficients attest to the high similarity between II ITP and II NP components and only moderate similarity between III ITP and I NP components.

**Table 2:** The Structure of Perception of AI Category in ITP and NP Groups: Loadings of Principal Components

Exemplars	Principal Components					
	ITP Group			NP Group		
	I	II	III	I	II	III
AR	.30	.69	−.11	.19	.61	.39
AV	.66	.21	.23	.35	.17	.32
Behavioral biometrics	.57	.14	.12	.48	.11	.31
Book writing program	.58	.11	.45	.57	.14	.37
Car navigation	.46	.44	.01	−.19	.50	.36
Chatbot	.65	.27	.29	.38	.14	.38
Chess program	.47	.45	.25	.46	.03	.42
Cobot	.00	.75	.14	.08	.79	.15
COVID-19 contact tracing apps	.16	.51	.14	.17	.39	.36
Cyborg	.00	.57	.33	.28	.63	−.12
Face generating program	.62	.08	.08	.52	.19	.38
Face replacing software	.70	.06	.10	.36	.09	.52
Facial recognition system	.70	.22	.23	.44	.13	.33
Financial service software	.58	.28	.24	.46	.12	.46
Go playing program	.68	.20	.29	.49	.01	.46
Humanoid robot	.20	.39	.70	.76	.31	−.12
Humanoid robot that creates images	.38	.20	.56	.67	−.01	.07
Image generation algorithm	.71	.13	.13	.38	.12	.57
Intelligent teaching/learning support system	.40	.52	.24	.17	.34	.51
IoT	.06	.66	.04	.18	.51	.30
License plate recognition software	.57	.42	−.10	−.02	.58	.43
Medical diagnostic software	.43	.34	.11	.10	.51	.47
Military robot	.18	.67	.19	.15	.72	.11
Music generation program	.44	.17	.39	.23	.09	.56
Nanobot	.17	.67	.36	.30	.67	−.02
Neural network	.45	−.12	.11	.18	−.25	.20
Poem-writing software	.59	−.04	.49	.57	−.04	.33
Police robot	.42	.19	.60	.63	.33	.08
Portrait-drawing robot	.33	.28	.55	.61	.23	.21
Press articles writing program	.64	−.07	.45	.60	.04	.40
Product recommendation software	.68	.07	−.14	.01	.03	.53
Program reconstructing images	.39	.44	.27	.43	.37	.12
Risk determining program	.66	.27	.17	.23	.31	.55
Robot/android priest	.13	.34	.70	.71	.22	−.06
Robotic animal	.14	.44	.56	.56	.36	−.01
Robotic child	.18	.46	.60	.71	.32	−.13
Sexbot	.22	.41	.39	.51	.34	.02
Skynet/Terminator	−.14	−.04	.47	.50	−.13	−.06
Social bot	.60	.06	.48	.62	−.01	.31
Social robot	.47	.31	.61	.57	.41	.21
Space robot	.13	.75	.17	.14	.77	.07
Strategy game program	.57	.17	.31	.58	.02	.40
Surgical robot	−.05	.72	.29	.16	.74	.06
System for debating	.56	−.01	.46	.64	−.08	.29
Text translation program	.57	.25	.03	−.11	.34	.68
UAV	.26	.65	.13	.13	.69	.16
Vacuum cleaning robot	.35	.63	.13	.03	.58	.37
Video recommendation algorithm	.74	.22	−.09	.08	.23	.72
Virtual therapist	.57	.27	.38	.67	.03	.25
VR	.02	.80	.01	−.04	.75	.22
Eigenvalue	10.91	8.43	6.00	9.22	7.72	6.10
Proportion of explained variance	21.8%	16.9%	12.0%	18.4%	15.4%	12.2%

*Note*. AV = autonomous vehicle; UAV = unmanned aerial vehicle; AR = augmented reality; IoT = internet of things; VR = virtual reality.

Loadings with absolute values of .50 or greater are printed in boldface.

The second component in both structures can be described as “supporting systems” (see Table 2). These are systems whose specific feature is making it easier for a person to go beyond their own limitations, as well as strengthening the ability to achieve goals in a difficult environment. The first component in ITP group, which explains the highest percentage (21.8%) of the variance of typicality scores, can be described as “algorithmic systems.” The exemplars are mainly autonomous softbots whose “engine” is hidden in an algorithmized “black box.” Partially similar, but much lower in the percentage of explained variance (12.2%), is the third component in the NP group. As in the case of ITP group, these are autonomous systems, but they refer to a narrower range of problems solved in the computer environment. These algorithms are utilitarian because they offer a new quality through data processing. That is why we refer to this factor as “useful programs.”

We called the third component in ITP group “robotic creatures.” The common feature of these specimens is their mechanical construction, which makes them similar to various individuals occurring in natural conditions, while allowing them to perform some of their functions. We used the label “creatures” because not all robots are humanoid (though in some ways all are animate), but they are examples of embodied AI.

The first factor in NP group explains the largest percentage (18.4%) of the variance of the typicality scores. We have defined it as “nature imitating systems.” It includes, among others, such exemplars as: humanoid robot, robotic child, virtual therapist, system for debating and social bot. These specimens differ in terms of design (robots and softbots), and their common feature is to imitate the functions assigned to living organisms. Artifacts grouped in this dimension are characterized by a high level of human-likeness. This is the result of work carried out in the developmental cybernetics (DC) and in the developmental robotics (DR) perspectives, which at the same time facilitate the perception of robot humanness and attribution of mental states (Manzi et al., 2020). Activities undertaken as part of DC focus on designing anthropomorphic physical and behavioral characteristics of robots, as well as simulating human mental processes and observing the dynamics of human-robot interaction. In turn DR focuses on the development of cognitive neural networks in the robot, leading to autonomous acquisition of complex cognitive functions. The example which reflects DC perspective in this factor is a robotic child and the DR research-debating system.

## Discussion

In our study, we looked for an answer to the question: What do people mean when they use a buzzword such as AI? In our research we identified a representation of AI in the ITP and NP minds. In the context of the obtained results our answer is as follows: It depends on the level of expertise of a given person. Despite the fact that there is no normative, universal systematization of the designations of the concept of AI, people manage to organize them using the knowledge they have, and this transpires both in the hierarchy of AI designates and in the dimensions of their grouping. The analysis of the obtained results allows to draw both theoretical and practical conclusions

The analyzes show the legitimacy of including the variable of IT specialist knowledge to psychological research on reception of new technologies. IT industry is one of the fastest-growing and it is in this area that the professions of the future are most often indicated. Our research shows that ITP and NPs can be described as experts and laypeople not only because of their professional experience, but also because of their subjective beliefs, the indicators of which are the higher self-esteem of knowledge and familiarity of AI exemplars. ITPs showed greater caution in terms of the overall assessment of the typicality of AI exemplars and the GOE scores in this group were not correlated with the familiarity scores, which was recorded in the case of NP. We can explain this if we accept that ITP and NP mean something different when assessing familiarity. The average reader will probably say without hesitation that Leo Tolstoy is a fine example of a writer, while literary specialists may hesitate to categorize him solely in such narrow terms and see him as a philosopher as well. It can be assumed that NPs combine knowledge of AI with popularity, which is consistent with the results of surveys in which respondents declare their knowledge of AI, although they are only slightly aware of the use of AI-driven products (Maison, 2019).

The significant influence of expertise in the categorization process was confirmed (Bailenson et al., 2002). We found similarities and differences between ITP and NP in mental representing of AI both in terms of the hierarchy of AI designates and the dimensions of their grouping. AV was placed at the top of AI categories both for ITP and NP, which may suggest the influence of the clear presence of this exemplar in the media. But while the GOE rating of AV was the highest, it is not the undeniable leader of the category. This is indicated by the similarity between GOE ratings of this object and three exemplars with lower ranks in both groups.

Based on the survey responses, our research shows that AI means primarily "visible AI," which is a far-reaching simplification. We found that highly rated by ITP exemplars of “invisible AI” had a lower rank in the graded structure identified in NP, and in the case of neural network this difference was extremely large (40). On the other hand, in the NP group, out of 15 assessed robots, which are examples of “visible AI” differing in human-likeness (e.g., social robot, cobot), as many as 13 had a higher rank than in the graded structure ITP, and the largest difference was related to the humanoid robot (15). ITPs seems to grasp AI more cognitively than sensually, which is understandable if you take into account the expertise of this group.

The overriding conclusion that ITP perceives AI rather as a “nervous system,” and NP “corporeality” cannot be sustained. The source of doubt is a more careful analysis of the graded structure of AI exemplars. The obtained results confirm that the prototype theories of concepts are insufficient to understand and fully describe the image of reality arising in the minds of people (Medin & Wattenmaker, 1987). While the taxonomies disclosed for ITP and NPs are based on three dimensions, their content is not identical. Specialist knowledge allows ITP to systematize AI exemplars based on both structural and functional elements. On the other hand, laymen refer rather to the functions that AI-driven products perform. For ITP, they are primarily algorithmic systems, while for NP they are systems that imitate the functions of living organisms. If we consider that the concept of intelligence means the ability to solve hard problems (Minsky, 1985), then we can assume that in the “AI” ITP emphasize the intelligence of artificial systems, while NP their artificiality. ITP primarily see in AI systems based on algorithms that, using mathematical models, are able to autonomously solve problems without human supervision. These systems, embodying AI defined as computational cognition (Rapaport, 2020), are undoubtedly for people professionally interested in digital technologies “core objects” of the category of AI. Classifying AV in this dimension suggests that for ITP it is primarily an example of an intelligent system, and only secondarily an AI-driven mechanism. On the other hand, fact, that for NP AI is mainly “nature imitating systems” corresponds to the understanding of AI as machines with minds. NP see in AI mainly the possibility of replacing the functions of various types of organisms, which confirms the relationships noticed in the graded structure. In this dimension, both robots and softbots are grouped as artificial forms of life that imitate the unique abilities of organisms. It can be said that intelligent algorithms do this as well, but according to the dichotomy between the cognitive and emotional abilities (Haslam, 2006) “nature imitating systems” reveal the abilities commonly associated with affect and intuition, which also applies to the robotic animal.

The practical conclusions of our research concern the sphere of education and communication. As it is known, for a biologist, a tomato will always be fruit, and for a consumer, a vegetable, although the problem will arise only when a biologist, when asked to buy fruit, brings a tomato grid. The initiative in the field of shaping knowledge about AI should be shown by teachers working with children, because they are sensitive to presence of the human-like physical features of robots (Manzi et al., 2020). This should shape an unbiased relationship with AI-based technologies from an early age. One way of doing it may be by incorporating elements of educational program at an early academic level, which Aoun (2018) called “humanics,” and proposed as a new academic discipline. “Humanics” extends old literacies (reading, writing, mathematics) by adding three new areas of literacy: data literacy, technological literacy, and human literacy. This learning model would include both machine understanding, data management, creative thinking, critical thinking, systems thinking, entrepreneurship, cultural agility and communication. The basis for unifying knowledge about AI may be the common dimension for the designation grouping of the concept of AI, which are “supporting systems,” for ITP and NPs. This is where the spaces for the systematization of AI exemplars of ITP representatives and NPs meet.

On this basis, activities supporting agreement and cooperation between the two groups can be designed. It is important for ITP to recognize that humanoid forms of little importance to them are the gateway to the world of AI for NPs. On the other hand, better knowledge of algorithms by NPs should reduce undesirable phenomena such as algorithm aversion, fear of AI, or even cyberparanoia strengthened by dystopian narrations. As we know “artificial intelligence,” as other buzzwords, is used more to impress or persuade than to inform. Thus sensational reports on the next possibilities of artificial intelligence (AI) should be counterbalanced by factual explanations. The initiative, especially in the working environment, should be taken over by ITP, whose role has increased even more during the pandemic. Our research does not only show that it is necessary, but it also indicates some common ground for cooperation with NPs.

This study contains a few notable limitations. The research identified the most typical designata of the concept of AI, while the prototype of AI was not identified. This requires the use of a different research methodology, with participants comparing individual exemplars with each other, which is a difficult though not an impossible task to perform due to their number. The AI exemplars taken into account in the study can be critically referred to. The selection of stimuli in this type of research is always a difficult decision, especially as the number of AI exemplars increases. In subsequent studies artificial humans should be included, because their presence on social networks is becoming more and more visible. For more in-depth analyzes, it seems important to collect detailed data on the sources of information on AI used by the respondents. This way it will be possible to define AI-narratives significant for the knowledge structure more precisely. Finally, this research was conducted during the first wave of the COVID-19 pandemic, when we saw a surge in interest in new technologies. In our research, we took into account the innovations that appeared on the market at that time (e.g., COVID-19 contact tracing apps), but the assessments of their typical nature were not graded highly in the structure of AI category. Nevertheless, we believe that it is worth treating our results as a benchmark for post-pandemic research.

## Supplementary Materials

For this article the following Supplementary Materials are available via PsychArchives (for access see Index of Supplementary Materials below)

- An appendix with the names of the exemplars being assessed in alpabetic order with photos given to the subjects

FortunaP.
GorbaniukO.
 (2022). Supplementary materials to "What is behind the buzzword for experts and laymen: Representation of “artificial intelligence” in the IT-professionals’ and non-professionals’ minds"
[Appendix]. PsychOpen. 10.23668/psycharchives.6530
PMC963254836348697

## Data Availability

Data for this article is freely available (see the Supplementary Materials  section).

## References

[sp1_r1] Fortuna P. Gorbaniuk O. (2022). Supplementary materials to "What is behind the buzzword for experts and laymen: Representation of “artificial intelligence” in the IT-professionals’ and non-professionals’ minds" [Appendix]. PsychOpen. 10.23668/psycharchives.6530 PMC9632548 36348697

